# Parkin the bus to manage stress

**DOI:** 10.15252/emmm.201910968

**Published:** 2019-07-17

**Authors:** Amandeep Kaur, Elizabeth E Gardiner

**Affiliations:** ^1^ ACRF Department of Cancer Biology and Therapeutics John Curtin School of Medical Research Australian National University Canberra ACT Australia

**Keywords:** Metabolism, Neuroscience

## Abstract

Autophagy, the process by which damaged or potentially cytotoxic cytosolic components are removed and destroyed by lysosomes, occurs to varying extents in all cells. Mitophagy describes an autophagic response that specifically targets damaged cytotoxic mitochondria for removal. This aggressive defense‐first policy (“*parking the bus*” in footballing terms) serves to protect the intracellular environment from cytotoxic mitochondrial components and maintain intracellular homeostasis. While mitophagy pathways have been extensively studied (Harper *et al*, 2018), precisely how the selective removal of a damaged mitochondrion is achieved in healthy cells, as well as in cells exposed to high oxidative stress conditions, remains unclear. Work from Lee and colleagues (Lee *et al*, 2019) has evaluated the molecular basis of mitophagy in platelets and has outlined some new molecular events that help control this process.

Platelets orchestrate the coagulation pathways that generate thrombin necessary for efficient thrombus (blood clot) growth and stability. To do this, platelets use mitochondrial‐driven cell death pathways to expose negatively charged phospholipids on the platelet membrane. Upon receptor‐driven activation, the cytosolic and mitochondrial calcium levels increase, which elevates phosphatidylserine exposure, providing a highly reactive surface conducive to activation of the contact pathway (O'Donnell *et al*, 2019). Reactive oxygen species (ROS) generated via platelet receptor engagement (Qiao *et al*, 2018) and calcium levels positively promote mitochondrial permeability transition pore (MPTP) opening, releasing mitochondrial components including cytochrome C in a process which is essential for both platelet activation and apoptosis (Choo *et al*, 2017; Fuentes *et al*, 2019). Because of the continual exposure of platelets to oxidative stress and mitochondrial damage, and the immediate implications for platelet lifespan if left unchecked, mitochondrial health and mitophagy are stringently monitored and controlled in platelets.

The roles of mitochondria in any cell type, including in platelets, comprise management of ROS, smooth and correct assembly of mitochondrial respiratory complexes for ATP synthesis through the mitochondrial electron transport chain, and maintenance of mitochondrial DNA. With regard to ROS, mitochondria control ROS clearance through assembly of phagosomes, serving to limit oxidative damage to lipids, nucleic acids, and proteins. The microtubule‐associated protein light‐chain 3 (LC3) is a ubiquitous marker of autophagy and is critical for the assembly of the phagosome (Fig 1). A range of mitochondrial membrane proteins harbor binding motifs that can bind to LC3 and promote mitophagy in damaged mitochondria. Platelet mitophagy requires Parkin, a mitochondrial E3 ubiquitin ligase, but to date, there has been limited information on this molecular process. Lee *et al* (2019) wondered whether selective mitophagy is controlled by specific LC3‐binding proteins, and identified the mitochondrial matrix enzyme methionine sulfoxide reductase (Msr) B2 which contains three LC3 binding motifs, as an LC3‐binding partner by mass spectrometry. In a series of elegant studies using mouse and human platelets as well as cell lines, the authors showed that MsrB2 was released via the MPTP from damaged mitochondria where it could then bind to Parkin, reducing and activating Parkin, preventing Parkin aggregation, and generating a polyubiquitinated form of MsrB2. This permitted the formation of a complex between Parkin, MsrB2, and LC3. Knockdown of MsrB2 using short interfering RNA approaches, or using platelets from a mouse with platelet‐specific MsrB2 deficiency, led to accumulation of inactive Parkin, loss of complex‐associated LC3, reduced mitophagy, and significant increases in pro‐apoptotic markers. Treatment of mice on high fat diet or cell lines with a cell penetrant peptide with sequence matching the MsrB2/LC3‐binding motif, designed to disrupt MsrB2/LC3 binding, also led to accumulation of Parkin, LC3, and MsrB2 and onset of apoptosis. From this work, the authors deduced that MsrB2, released only from mitochondria that were earmarked for destruction, was behaving as a molecular switch and triggering selective mitophagy.

**Figure 1:**
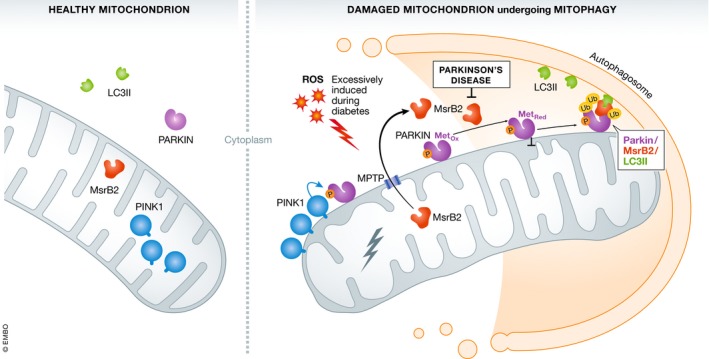
Molecular steps in mitophagy Activation of Parkin is mediated by the accumulation of mitochondrial outer membrane protein kinase PINK1, and oxidation of Parkin which subsequently reduced by MsrB2 that has been released from a damaged mitochondrion. Ubiquitination of MsrB2 by Parkin would then proceed, enabling the formation of the Parkin/MsrB2/LC3II complex and the autophagosome leading to mitophagy. This pathway signals the destruction of a damaged mitochondrion and is accentuated in environments with high levels of ROS (like in diabetes), while reduced in Parkinson's disease.

Platelets are a convenient source for analyzing mitochondrial function changes of patients with type 2 diabetes (DM; Avila *et al*, 2012). The loss of an appropriate mitophagy response in DM platelets results in failure to protect against oxidative stress and can lead to increased thrombosis (Lee *et al*, 2016). Platelet hyperactivity is a hallmark of diabetes, which contributes to the increased risk of thrombotic events (heart attack, stroke) seen in these patients. Lee and colleagues examined platelets from DM patients and detected increased levels of MsrB2 when compared to platelets from healthy donors. They also observed MsrB2 was co‐localized with LC3 in co‐immunoprecipitation experiments and by confocal and electron microscopy. Under oxidative stress, DM platelets commonly display evidence of mitophagy in greater excess compared to healthy donor platelets. Intriguingly the authors also present data suggesting that this pathway is dysfunctional in platelets from patients with Parkinson's disease (PD) and demonstrate selective reduction in MsrB2 and LCII, and increased apoptosis in PD platelets. Elimination of aberrant mitochondria is a crucial process in age‐related neurodegenerative disorders, such as Parkinson's disease (PD) and Parkin mutations have been implicated in autosomal recessive juvenile Parkinsonism (Miller & Muqit, 2019). More recently, a role for Parkin as a tumor suppressor gene has emerged (Wahabi *et al*, 2018), underscoring the importance of mitophagy regulation in cell biology. Taken together, the data obtained using platelets from both DM and PD patients to demonstrate the role of MsrB2 and this pathway in pathophysiological contexts have helped explain the exquisite organelle‐specific selectivity of mitophagy. Undoubtedly, future research focused on unraveling the shared and divergent Parkin‐mediated pathways in PD and cancer will provide insights into these disease processes and potential therapeutic avenues for the management of oxidative stress in a host of disease states (Miller & Muqit, 2019).
